# X-Ray Diagnosis of Disease of Stomach

**Published:** 1915-02

**Authors:** 


**X-Ray Diagnosis of Disease of Stomach.** Donald McGavin of Wellington, New Zealand Med. Jour., Oct. 1914, classifies the substances introduced to facilitate X-ray examination as more and less permeable than the tissues. The former are air pumped in (or swallowed) and carbon dioxid developed chemically and are used in a single condition—carcinoma of the cariac portion, brought into relative shadow by inflation. In discussing the latter, the following points are made: bismuth subnitrate poisonous, carbonate and oxy-chlorid non-poisonous; pure barium carbonate preferred as cheaper, other salts must not be used as they are toxic; Baradiol, a German preparation contains barium sulphate with carbohydrate ready for suspension; zirconim oxid, 30-50 grams, may be used, especially in children who do not tolerate barium well; cerium, cerium thorium, thorium dioxid and oxid of iron, as in combination with chocolate in what is called diaphanite, may also be employed to give a shadow. By using a heavy and a light, swimming capsule, the emptying time may be determined by the fact that when the stomach is empty, both capsules will lie side by side at the bottom of the stomach. Swimming capsules also show the depth of parasecretion. Capsules with soluble envelopes show the action of gastric juice. (But many supposedly soluble capsules require an hour to melt. Ed.) Hollow sounds are no longer used. Always screen before administering the opaque substance, to exclude other shadows. The screen should be about 30x40 c.m. Use a hard tube of 10-12 c.m. parallel spark, secondary current of 2 milliampers, a good movable stative with iris diaphragm. In taking plates focus for spine of 6th dorsal vertebra and expose for less than one second, while the patient holds his breath in deep inspiration, always in the upright position. Good peristalsis with failure to empty may be due to pyloric spasm which may be relieved by injecting atropine or 5 c.c of papaverine. The author speaks somewhat sceptically of diagnosing for or against ulcer. Bismuth does not necessarily remain on a denuded surface and may remain in folds of mucous membrane without ulcer.

